# Spatiotemporal distribution of neural crest cells in the common wall lizard *Podarcis muralis*

**DOI:** 10.1002/dvdy.758

**Published:** 2024-11-19

**Authors:** Robin Pranter, Nathalie Feiner

**Affiliations:** ^1^ Department of Biology Lund University Lund Sweden; ^2^ Max Planck Institute for Evolutionary Biology Plön German

**Keywords:** cell migratory routes, gene expression patterns, HNK‐1, squamate reptiles

## Abstract

**Background:**

Neural crest cells (NCCs) are migratory embryonic stem cells that give rise to a diverse set of cell types. Here we describe the dynamic distribution of NCCs in developing embryos of the common wall lizard *Podarcis muralis* inferred from 10 markers. Our aim is to provide insights into the NCC development of lacertid lizards and to infer evolutionary modifications by comparisons to other tetrapods.

**Results:**

NCC migration is ongoing at oviposition, following three streams in the head and multiple in the trunk. From 21ss, we observe expression patterns indicating the beginning of differentiation toward mesenchymal and neuronal fates. By 35ss, migration is restricted to caudal levels, and fully differentiated chromaffin cells are observed.

**Conclusions:**

We find that some markers show patterns that differ from other tetrapods. For example, the antibody HNK‐1 labels three NCC streams from the hindbrain while some comparable reptile studies describe four. However, the information emerging from all markers combined shows that the overall spatiotemporal distribution of NCCs in the common wall lizard is largely conserved with that of other tetrapods. Our study highlights the dynamic nature of seemingly canonical marker genes and provides the first description of spatiotemporal NCC dynamics in a lacertid lizard.

## Key Findings

Integrating the insights gained from eleven NCCsmarkers, we show that:

- The overall behaviour of NCCs in the common wall lizard is highly conserved with that described for other tetrapods.
- Nevertheless, we find that some seemingly canonical marker genes show expression patterns that are distinct from those previously reported for other tetrapods.
- We do not find support for two previously described aberrant patterns of NCC behaviour in snake or chameleon in the common wall lizard, indicating that these patterns are likely not common to squamate reptiles.

## 1 INTRODUCTION

Neural crest cells (NCCs) are transient, migratory stem cells that are unique to vertebrates and that give rise to many different cell types, thus contributing to a diversity of traits. NCCs are specified in the dorsal neural tube, from where they delaminate in an epithelial‐to‐mesenchymal transition (EMT) and subsequently migrate throughout the embryo and differentiate. The cell types they give rise to include chromatophores responsible for skin color, osteocytes and chondrocytes that constitute the facial skeleton, neurons, and glia of the peripheral nervous system and endocrinal chromaffin cells in the adrenal gland.^1^

NCCs originated at the dawn of vertebrates, and many of the vertebrate key innovations (most notably the jaw and other craniofacial structures) develop from NCCs.^2^ For this reason, a substantial body of research has targeted the evolutionary origin of NCCs by investigating NCCs in early branching vertebrates and NCC‐like cells in non‐vertebrate chordates.^3^, ^4^, ^5^ In addition to these attempts to elucidate the evolutionary origin of NCCs in a macro‐evolutionary context, much research has focused on investigating their function and development in a number of model organisms, most notably mouse (*Mus musculus*), chicken (*Gallus gallus*), African clawed frog (*Xenopus laevis*), and zebrafish (*Danio rerio*). Motivation for this research partially stems from the fact that failure of NCC functions causes neurocristopathies, a highly heterogeneous family of congenital diseases,^6^ which makes them relevant from a medical perspective.

In squamate reptiles (lizards and snakes), NCC biology has been investigated in three species: the California kingsnake (*Lampropeltis getula californiae* ^7^), the Egyptian cobra (*Naja haje haje* ^8^), and the veiled chameleon (*Chamaeleo calyptratus* ^9^). The reports on the two snake species mainly focus on the migration of NCCs in the trunk, while the report in chameleon provides a comprehensive description of both cranial and trunk NCCs. Both snakes and chameleons have highly derived body plans including in NCC‐derived traits such as skull morphology.

In the chameleon, cranial NCC migration starts between somite stage 4 and 6 (4ss and 6ss),^9^ which is similar to mouse (5ss^10^, ^11^, ^12^), human (4ss^13^) and chicken (6ss^14^ ^[p66]^). In general, cranial NCC migration typically follows three main dorsolateral streams.^15^ The NCCs in the anterior‐most stream are specified in the caudal midbrain and the first three rhombomeres of the hindbrain and contribute cells to the rostrum, maxillary process, and first pharyngeal arch.^15^ The second stream is constituted by NCCs that are specified in rhombomeres three through five (anterior to the otic vesicle) and migrate to the second pharyngeal arch.^15^ And lastly, the NCCs in the third stream are specified in rhombomeres five and six (posterior to the otic vesicle) and migrate to the third pharyngeal arch.^15^ In the chameleon, however, Diaz et al.^9^ identified a fourth stream of cranial NCCs starting posteriorly to the third stream and migrating to the fourth pharyngeal arch. An equivalent stream has also been described in alligator (*Alligator mississippiensis*) and ostrich (*Struthio camelus*).^16^

NCC migration in the trunk follows multiple, periodic streams starting in the dorsal neural tube. Viewed in cross‐sections in a transverse plane, these streams typically follow two different routes.^17^, ^18^, ^19^ An early wave of migrating cells follows a ventromedial route traversing between somites and through the anterior portion of somites and giving rise to, for example, peripheral nervous system and chromaffin tissue.^17^, ^18^, ^19^ A later wave follows a dorsolateral route and gives rise to chromatophores.^17^, ^18^, ^19^ While this pattern seems to be largely conserved across tetrapods,^7^, ^8^, ^9^ it has been reported that some NCCs in the second wave follow a ventromedial rather than dorsolateral route in the California kingsnake.^7^ The equivalent was not reported for chameleon or cobra.^8^, ^9^

In between and partially overlapping with the cranial and trunk NCCs, there is a small subpopulation of NCCs called vagal NCCs.^15^ Similar to trunk NCCs, vagal NCCs migrate along both dorsolateral and ventromedial routes.^20^ The dorsolateral migrating NCCs reach the pharyngeal arches and the heart.^20^ Some vagal NCCs of both the dorsoventral and ventromedial streams reach the foregut which they then follow caudally.^20^ Vagal NCCs have not been described in squamates.

While efforts in squamates have increased our understanding of NCC specification, migration, and differentiation across vertebrates, evolutionary changes in NCC behavior and potentially associated phenotypic changes are currently underexplored.^21^ Since NCCs give rise to functionally diverse and ecologically relevant traits ranging from coloration (e.g., pigment cells) and morphology (e.g., skull and jaw bones) to physiology and behavior (e.g., adrenal gland influencing aggression), their coupling in a shared developmental origin can potentially have evolutionary consequences. Indeed, this coupling has been suggested to explain the domestication syndrome, which is based on the observation that domesticated animals share a set of changes in NCC‐derived traits.^22^, ^23^ In general, due to their central role during development, modifications in the regulation of NCCs are expected to have the potential to generate biases in micro‐evolutionary variation in natural populations of vertebrates and therefore contribute to adaptation and diversification.^21^, ^24^, ^25^ To evaluate hypotheses like this, establishing patterns of NCC migration in species where NCCs may be implied in micro‐evolutionary adaptation is crucial.

Here we present a description of NCCs in the common wall lizard (*Podarcis muralis*), which is a small (~50–70 mm snout‐to‐vent length^26^), egg‐laying lacertid. This species belongs to a genus of lizards distributed in the Mediterranean and Southern/Central Europe that shows high variation in coloration and color patterns.^27^, ^28^ Within the common wall lizard, there is substantial variation in predominantly NCC‐derived traits, namely, coloration (ranging from brown/tan to black/green),^29^, ^30^, ^31^, ^32^ social behavior (in particular aggression in male–male interactions),^33^, ^34^ and morphology (e.g., body size, head size, and shape).^29^, ^32^ In Italy, the strength of expression of these traits is tightly correlated across the landscape, culminating in the *nigriventris* syndrome at one end of the extreme and the ancestral phenotype at the other end.^32^ The genetic basis of these trait differences has been identified as being polygenic, with more than half of all identified genes having a known association with the regulation of NCCs.^32^ This prompted us to investigate NCC biology in this species. Females typically lay 2–3 clutches per breeding season with 2–10 eggs per clutch.^26^ ^(p189)^ We use a set of 10 NCC markers that target different aspects of NCC biology to investigate the spatial distribution and infer migration patterns of NCCs from the earliest stage at oviposition (~13ss) until the latest stages of NCC activity (~ 64ss). To identify evolutionary changes to the spatiotemporal distribution of NCCs, we compare our observations to equivalent patterns described in other squamates and more distantly related tetrapods. We find broad conservation in general NCC specification, migration, and differentiation, but also taxonomic differences in the expression patterns of individual markers. Taken together, our description of spatial and temporal patterns of NCCs during embryonic development expands our understanding of the taxonomic diversity in NCC biology and presents a first step toward investigating the role of NCCs in shaping micro‐evolutionary patterns.

## 2 RESULTS AND DISCUSSION

Given that NCCs are a heterogeneous and highly dynamic cell population, there is no single marker that distinguishes them from other cell types throughout development. We therefore use a two‐pronged approach to resolve spatiotemporal dynamics of putative NCCs. First, we use immunostaining with antibodies against Human Natural Killer 1 (HNK‐1) to label putative NCCs throughout development. HNK‐1 is a carbohydrate on the cell surface of NCCs that is involved in cell–cell communication during migration^35^, ^36^ and it is a widely used marker of newly formed and migrating NCCs^16^, ^17^, ^37^, ^38^, ^39^, ^40^, ^41^, ^42^, ^43^ (note however that it is not labeling NCCs in mouse).^44^ This antibody has been successfully applied in other squamate species,^7^, ^8^, ^9^ which allows a straightforward comparison between our observations in the common wall lizard and other squamates.

Since HNK‐1 is known to also label other cell types, in particular at late developmental stages,^45^ we complement HNK‐1 immunostainings with gene expression analyses of NCC marker genes. For this second approach, we select a panel of nine marker genes whose expression patterns are visualized using in situ hybridizations. Collectively, these marker genes label the majority of NCC subpopulations and target various stages of NCC development.^46^ Note however that some markers do not exclusively label NCCs since they fulfill pleiotropic functions (e.g., *Twist1* stains mesodermal cells in addition to NCCs^47^). Specifically, we select *Wnt1* and *Snai2* to label predominantly early NCCs, *Snai1*, *Twist1*, and *Sox9* to mark NCCs biased toward mesenchymal fates and *FoxD3*, *Sox10*, and *Phox2b* to mark NCCs biased toward neuronal and glial fates.^46^, ^48^, ^49^ In addition, we use *Tyrosine hydroxylase* (*Th*) to label NCCs differentiating into chromaffin cells.^50^ While HNK‐1 immunostaining and in situ hybridizations provide snapshots of the location of NCCs throughout development, we infer migratory routes from the series of observed imaging data aided by comparisons to relevant literature. For clarity, results for cranial, vagal (where applicable), and trunk NCCs are presented separately for each marker.

### 2.1 HNK‐1 stains migratory and early differentiating NCCs

**FIGURE 1:**
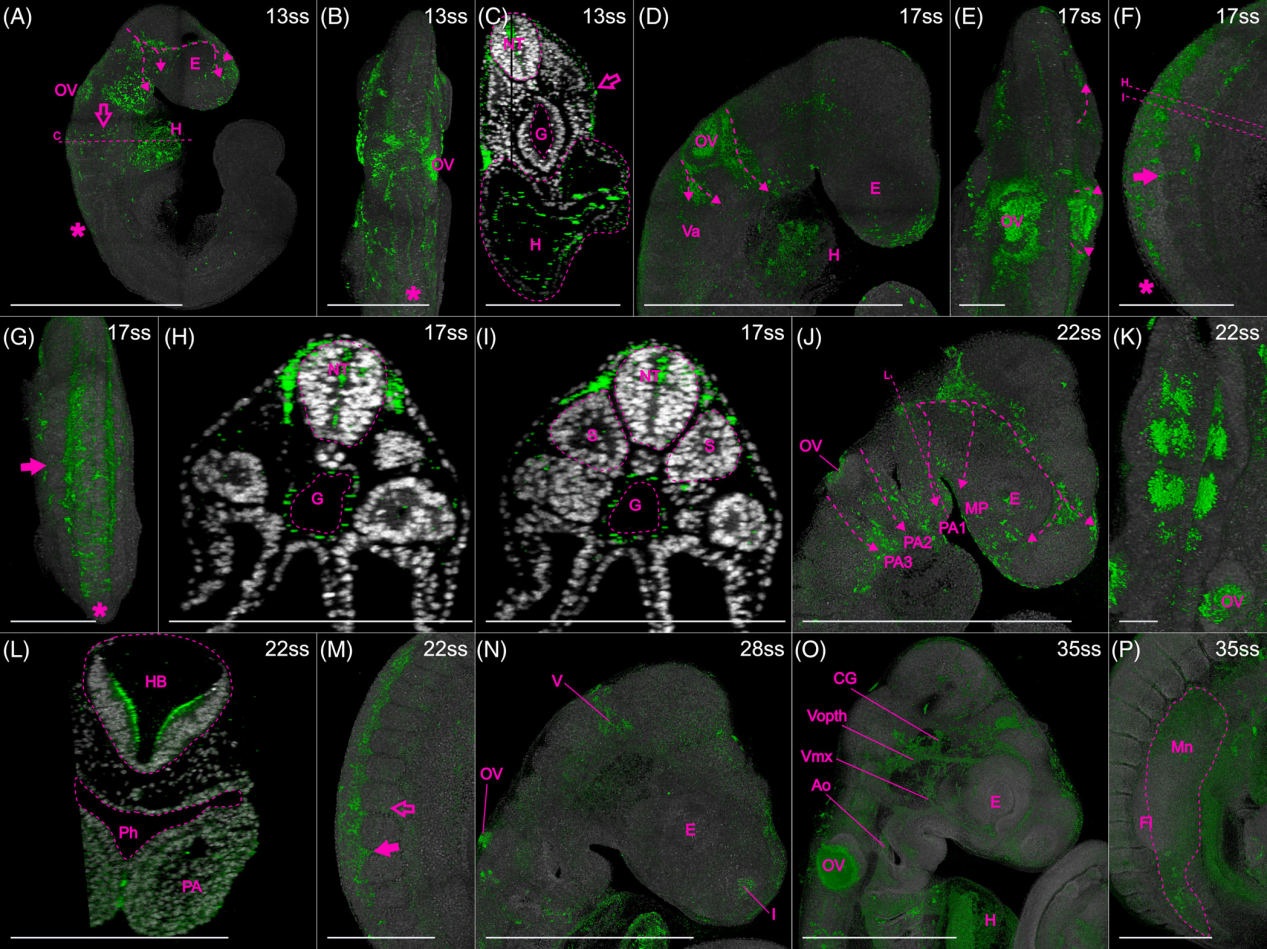
Immunohistochemistry of HNK‐1 stains migratory and some early differentiating NCCs in common wall lizard embryos. Dashed arrows indicate inferred cranial NCC migration. (A–C) 13ss embryo in lateral (A) and dorsal (B) view, and in optical cross‐section (C). Asterisks indicate expression in premigratory and early migratory NCCs, and open arrows indicate vagal NCCs. Staining in the heart is best seen in (A) and (C). (D,E) Head of 17ss embryo in lateral (D) and dorsal (E) view. (F–I) Trunk of 17ss embryo in lateral (F) and dorsal (G) view, and in optical cross‐sections (H,I). Arrows indicate inter‐somitic NCC migration and asterisks indicate premigratory and early migratory NCCs. (J–L) Head of 22ss embryo in lateral (J) and dorsal (K) views, and in optical cross‐section (L). (M) Trunk of 22ss embryo in lateral view. Arrow indicates inter‐somitic NCC migration and open arrow indicates migration at the level of a somite. (N,O) Heads of 28 (N) and 35ss (O) embryos in lateral view. (P) Trunk of 35ss embryo in lateral view. (O) and (P) were rendered with lower DAPI‐opacity. The locations of the optical cross sections (C), (H), (I), and (L) are indicated in (A), (F), and (J). E, eye; Fb, forebrain; Fl, Forelimb; G, gut; H, heart; HB, hindbrain; Mn, mesonephros; MP, maxillary process; NT neural tube; OV, otic vesicle; PA, pharyngeal arch; Ph, pharynx; S, somite; Va, vagal. Cranial nerves: I, olfactory nerve (also known as cranial nerve 1); V, trigeminal nerve (also known as cranial nerve 5); Vmx, maxillary branch of the trigeminal nerve; Vopth, ophthalmic branch of the trigeminal nerve. Scale bars in (B–D), (F–L) and (O–P) are 0.5 mm. All other scale bars are 1 mm.

HNK‐1‐positive cells are present in all investigated stages (five stages ranging from 13ss to 35ss; Figure 1A–P). Cranial NCC migration can be inferred along three routes. Each stream originates in the dorsal midline and extends ventrally in the developing head (Figure 1A,B,D,E,J; dashed arrows indicate inferred streams). The first stream starts at the level of the midbrain–hindbrain boundary and reaches toward the rostrum, maxillary process, and the first pharyngeal arch (Figure 1A). The second and third inferred streams originate in the hindbrain on either side of the otic vesicle, extending toward the second and third pharyngeal arches, respectively (Figure 1D,E). While the first stream is visible already at the first investigated stage (13ss), the second and third streams are seen more clearly from 17ss. The three streams of migrating cranial NCCs remain recognizable until 22ss (Figure 1S), after which migration of cranial NCCs presumably seizes. By then, HNK‐1 signal is widely spread in the developing head including structure that will give rise to the facial skeleton such as the rostrum, maxillary process, and the first three pharyngeal arches (Figure 1J), and some cranial placodes (Figure 1N), which are co‐derived from NCCs and placodal cells.^51^, ^52^ HNK‐1 signal in the cranial peripheral nervous system is maintained as the cranial placodes give rise to cranial nerves (Figure 1O; cranial nerve nomenclature follows Diaz et al. (2019),^9^ Lee et al. (2003)^52^ and Streeter (1905)^53^). There is also strong segmental staining in the rhombomeres of the hindbrain (Figure 1K,L).

At vagal levels, NCC migration can first be inferred by 13ss along a dorsolateral route to the heart (open arrows in Figure 1A,C), which is also strongly stained by HNK‐1 (indicated with “H” in Figure 1A,C,D). At later developmental stages, there is a branch from the third cranial stream extending caudally into the trunk, which potentially indicates a stream of vagal NCCs on their way to populate the gut and gives rise to the enteric nervous system (indicated with “Va” in Figure 1D). Consistently, cross‐sections of the trunk reveal staining around the gut (Figure 1H,I); a similar staining has been shown in chicken.^38^

In the trunk, HNK‐1 signal in NCCs can be roughly divided into four stages: premigratory, early migratory, migratory, and early neural differentiation. HNK‐1 signal is present in a stripe along the dorsal midline where premigratory NCCs are expected (Figure 1B,E,G). Immediately lateral to the dorsal midline, HNK‐1 stains early migratory NCCs in loose cell aggregates, not yet forming migratory streams (Figure 1A,B,F,G,M). The staining of premigratory and early migratory NCCs is found at progressively more posterior positions along the trunk as development proceeds. At more anterior levels, multiple streams of NCCs can be inferred between the somites (arrows in Figure 1F,G,M), and, to a lesser extent, at the level of the somites (open arrow in Figure 1M). Cross‐sections reveal that the streams of migratory NCCs take a ventral route between somites (inter‐somitic; Figure 1H), while they take a dorsolateral route at the levels of the somites (Figure 1I). NCC migration in the most caudal region of the trunk is still ongoing in the oldest investigated stages (28ss and 35ss). While HNK‐1 signal is known to mark the NCC‐derived dorsal root ganglia in chicken,^38^ and also a turtle (*Trachemys scripta*),^54^ an alligator (*Alligator mississippiensis*),^16^ and the Californian kingsnake (*Lampropeltis getula calilforniae*),^7^ this staining is absent in common wall lizards (Figure 1P). The reported HNK‐1 staining in dorsal root ganglia in chameleon is also less distinct than in other organisms.^9^ This emphasizes that HNK‐1 is neither specific nor general enough to figure as a sole NCC marker, and therefore requires complementation with other markers. In later stages, there is some HNK‐1 staining in the mesonephros (Figure 1P). This is also seen in other reptiles, but it is unclear if it is due to NCCs contributing to this organ.^7^, ^9^ At later stages (35ss), we detect the HNK‐1 signal in blood cells inside the aorta and heart (Figure 1O). Presumably, this is caused by the fact that blood cells possess HNK‐1 on their cell surface, despite not being NCC‐derived.^45^

### 2.2 *Wnt1* and *Snai2* are expressed in premigratory NCCs

Besides being a crucial organizer of the midbrain–hindbrain region,^55^ *Wnt1* is a classic marker of the neural border (including the rhombic lips^56^) and inducer of NCC specification.^46^, ^57^ And the *Wnt1‐Cre* transgenic lines have been instrumental in numerous experimental studies of NCCs in mice,^48^, ^58^, ^59^ highlighting their crucial role as a NCC regulator.

**FIGURE 2:**
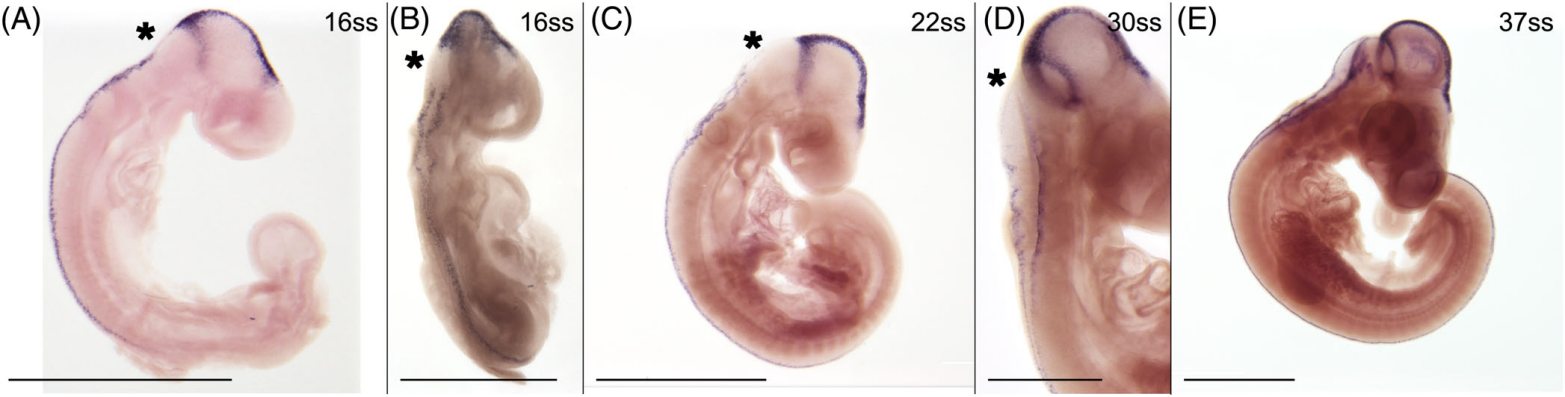
*Wnt1* is expressed along the dorsal midline. Asterisks indicate a gap with no expression in the anterior hindbrain. (A,B) 16ss embryo in lateral (A) and dorsolateral (B) view. (C) 22ss embryos in lateral view. (D) Hindbrain of 30ss embryo in dorsolateral view. (E) 30ss embryos in lateral view. Scale bars in (B) and (D) are 0.5 mm. All other scale bars are 1 mm.

*Wnt1* is expressed in all investigated stages (10 stages ranging from 13ss to 60ss), mostly along the dorsal midline (Figure 2A–E). Through most of the investigated developmental stages (16–30ss), there is a noticeable gap of expression in the most anterior part of the hindbrain (the metencephalon; asterisks in Figure 2A–D), separating the expression into an anterior and a posterior domain. This pattern has been described in chicken.^55^ The gap of expression overlaps with the origin of the first cranial stream, as inferred from HNK‐1 staining.

The anterior domain runs along the dorsal midline of the head from the level of the diencephalon to the midbrain–hindbrain boundary (Figure 2A,C). It has some lateral extensions that are presumably not NCC related and will not be described in further detail. The posterior domain consists of two parallel lines running along the rhombic lips in the hindbrain (Figure 2D) and continuing caudally along the dorsal midline of the trunk (Figure 2B), extending caudally approximately as far as to the last formed somite pair, and continuously extends posteriorly as development progresses (Figure 2A,C,E). The caudal extension of *Wnt1* expression is concordant with premigratory NCCs, as inferred from HNK‐1 staining.

*Snai1* and *‐2* are classical NCC markers previously known as *Snail* and *Slug*.^60^ They are transcription factors that control cadherin transcription and thereby EMT.^46^ There was some uncertainty in regards to the homology relationships between these genes, and EMT is induced by paralogous genes in chicken (*Snai2*) and mouse (*Snai1*).^61^ We confirm the expected orthology relationships between *Snai1* and ‐*2* of the common wall lizard and their homologs in human, mouse, and chicken (see Methods) and investigate both of them using in situ hybridization. *Snai2* expression is investigated in eight stages ranging from 13ss to 51ss.

**FIGURE 3:**
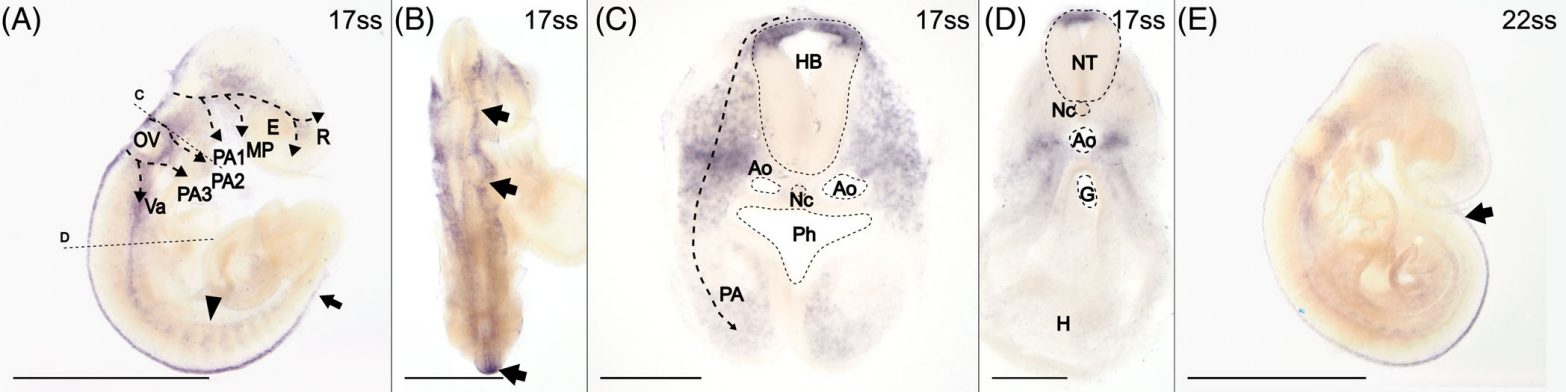
*Snai2* is expressed in both premigratory and migratory NCCs. Arrows indicate expression along the dorsal midline. (A–D) 17ss embryo in lateral (A) and dorsal (B) views and in cross‐sections (C and D) at the levels indicated in (A). Arrowhead indicates migration through the anterior half of the somites and dashed arrows indicate the inferred streams of migratory cranial NCCs. (E) 22ss embryo in lateral view. Ao, aorta; E, eye; G, gut; H, heart; HB, hind brain; MP, maxillary process; Nc, notochord; NT, neural tube; OV, otic vesicle; PA, pharyngeal arch; Ph, pharynx; R, rostrum; Va, vagal NCCs. Scale bar in (B) is 0.5 mm and scale bars in (C) and (D) are 0.1 mm. All other scale bars are 1 mm.

*Snai2* expression in the head is found in putative premigratory NCCs along the dorsal midline and the rhombic lips in the hindbrain (Figure 3A,B), and in the three streams of cranial NCC migration previously inferred with HNK‐1 staining (dashed arrows in Figure 3A,C). In cross‐section through the second stream, *Snai2* expression can be seen from the dorsal tip of the neural crest to the pharyngeal arches (Figure 3C). While a caudally migrating vagal stream of NCCs is only weakly suggested by HNK‐1, it is strongly marked by *Snai2* expression, visible as a branch from the third cranial stream (Figure 3A). In cross‐section at the level of the heart, strong *Snai2* expression is located lateral to the gut, presumably indicating caudally migrating vagal NCCs (Figure 3D).^20^

Similar to HNK‐1, *Snai2* expression in putative premigratory NCCs along the dorsal midline of the trunk progressively extends caudally as development progresses (arrows in Figure 3A,E). *Snai2* expression is also evident in multiple streams of migratory NCCs traversing the somites (Figure 3A).

Taken together, the expression patterns of *Wnt1* and *Snai2* are compatible with the expected location of premigratory NCCs. As in chicken, but not mouse, *Snai2* seems to be expressed where NCCs are expected to undergo EMT.^61^ Similar to HNK‐1, *Snai2*, but not *Wnt1*, is expressed in migratory NCCs. In addition, *Snai2* expression labels putative vagal NCCs more clearly than HNK‐1.

### 2.3 *Twist1*, *Snai1*, and *Sox9* are expressed in NCCs biased toward mesenchymal fate

*Twist1* is a well‐known NCC marker and transcription factor that has been studied in various other organisms. It has been found to be involved in EMT,^62^ maintenance of a migratory state, repression of neuronal differentiation,^63^ and induction of chondrocyte differentiation.^48^

**FIGURE 4:**
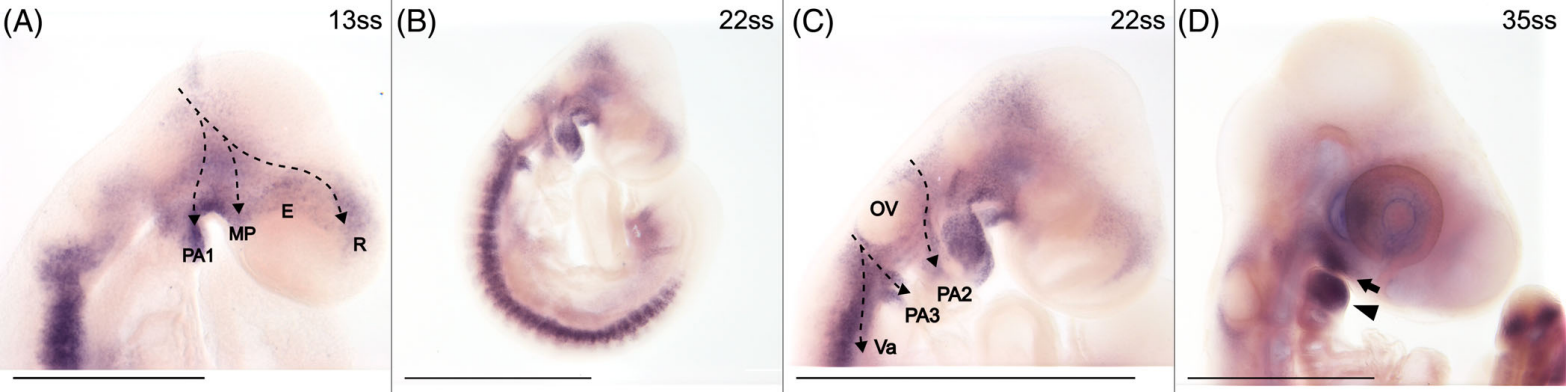
*Twist1* is expressed in migratory cranial NCCs. (A–C) Heads of 13, 17, and 22ss embryos in lateral view. Dashed arrows indicate the inferred streams of cranial NCC migration. (D) Head of 35ss embryo in lateral view. Arrow indicates the maxillary process and arrowhead indicates the first pharyngeal arch. E, eye; MP, maxillary process; OV, otic vesicle; PA1‐3, pharyngeal arch 1–3; R, rostrum; Va, posterior stream of vagal NCCs. Scale bar in A is 0.5 mm and all others are 1 mm.

Expression of *Twist1* is observed in all investigated stages (7 stages ranging from 13 through 51ss; Figure 4A–D). Cranial *Twist1* expression is missing along the dorsal midline but is consistent with the expected location of migratory NCCs (including the vagal branch) observed with HNK‐1 and *Snai2* (dashed arrows in Figure 4A,C). The lack of *Twist1* expression in the presumed premigratory NCCs in the dorsal midline is consistent with findings in mouse and chicken,^63^, ^64^ but different from frog and zebrafish.^62^ Later expression is mostly restricted to the maxillary process and pharyngeal arches (arrow and arrowhead in Figure 4D). Given the described role of *Twist1* as an inducer of chondrocytes,^48^ this expression likely labels the formation of craniofacial structures in these regions. At early stages, there is also broad but transient expression of *Twist1* in the head ectoderm. While *Twist1* is also extensively expressed in the trunk, it is restricted to putative mesodermal cells in the somites and limb buds and will therefore be disregarded here.

**FIGURE 5:**
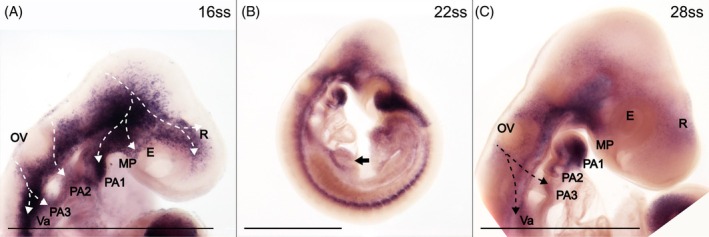
*Snai1* is expressed in migratory cranial NCCs. (A) Head of 16ss embryo in lateral view. Dashed lines indicate inferred streams of cranial NCC migration. (B) 22ss embryo in lateral view. Arrow indicates a bifurcation in the inferred caudal stream of vagal NCCs. Expression in the trunk is in tissues of non‐NCC origin. (C) Head of 28ss embryo in lateral view. The dashed arrows indicate the third stream of inferred cranial NCC migration. E, eye; MP, maxillary process; OV, otic vesicle; PA1‐3, pharyngeal arch 1–3; R, rostrum; Va, posterior stream of vagal NCCs. Scale bars are 1 mm.

*Snai1* is expressed in all investigated stages (7 stages ranging from 16ss to 51ss) and its expression patterns resemble that of *Twist1* (Figure 5A–C). In contrast to *Snai2*, *Snai1* is not expressed where premigratory NCCs are expected to undergo EMT in the common wall lizard (Figure 5A,B), which is different from findings in mouse but consistent with findings in chicken.^61^ Compared to Twist1, the different streams of cranial migration are not as easily discernible for *Snai1* since the intensity of the expression signal is generally high (Figure 5A). Given this high intensity, it is possible that not only NCCs contribute to the signal.

Together with *Snai1*, ‐*2* and *Twist1*, *Sox9* is known to be a crucial regulator of EMT^46^, ^65^ and it is also known to regulate migration and chondrocyte differentiation of cranial NCCs.^46^ However, in the trunk, *Sox9* is expressed in premigratory but not migratory NCCs in chicken.^66^

**FIGURE 6:**
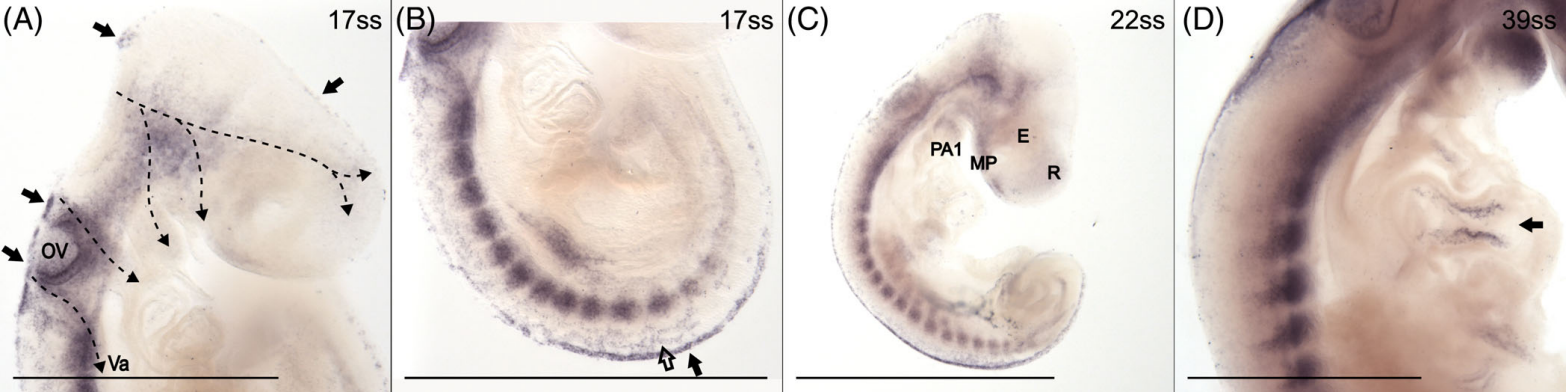
*Sox9* expression labels both premigratory NCCs and migratory cranial NCCs. (A) Head and (B) trunk of 17ss embryo in lateral view. Dashed arrows indicate inferred streams of cranial NCC migration. Arrows indicate expression in the dorsal midline and open arrow indicates early migratory NCCs. (C) 22ss embryo in lateral view. (D) Trunk of 39ss embryo in lateral view. Arrow indicates expression in the heart. E, eye; MP, maxillary process; OV, otic vesicle; PA, pharyngeal arch; R, rostrum; Va, posterior stream of vagal NCCs. Scale bars in (A) and (B) are 0.5 mm, and all others are 1 mm.

In the common wall lizard, *Sox9* is expressed in all investigated stages (8 stages ranging from 13ss through 51ss; Figure 6A–D). In the head, *Sox9* appears to label both premigratory (arrows in Figure 6A) and migratory (dotted arrows in Figure 6A) NCCs. In the third stream, it is mainly the caudally directed putative vagal branch that can be inferred. Later in development, the expression in the inferred streams extends to much of the surface of the head (Figure 6C). At this point, the expression pattern resembles that of the late *Twist1* expression.

*Sox9* expression in the trunk is consistent with premigratory NCCs along the dorsal midline, resembling that of *Snai2*. There is also expression in what appears to be early migratory NCCs just lateral to the dorsal midline that have not yet condensed into migratory streams (Figure 6B,C). Otherwise, *Sox9* does not appear to be expressed in migratory NCCs in the trunk, which is consistent with findings in chicken.^66^ There is expression in non‐NCC related structures (Figures 6B,C), which will not be described in more detail here. Some *Sox9* expression can be seen in the heart at 39ss (Figure 6D), where it stains a structure that may be the outflow tract, which is partially derived from NCCs in mouse.^63^

In summary, the expression patterns of *Snai1*, *Twist1*, and *Sox9* generally support the spatiotemporal location of migratory cranial neural crest cells inferred from HNK‐1. In addition, they distinctly label regions such as the maxillary process and pharyngeal arches where the facial skeleton will develop. *Sox9* is also expressed in the heart and in putative premigratory NCCs along the dorsal midline of both the head and trunk.

### 2.4 *Sox10*, *FoxD3* , and *Phox2b* are expressed in NCCs biased toward neuronal and glial fates

After HNK‐1, *Sox10* is arguably the most canonical NCC marker, and together with *FoxD3* and *Ets1*, it is considered a NCC specifier gene.^46^ It is a marker of migratory NCCs,^67^ and it is necessary for NCC differentiation into melanocytes, neurons, and glia.^46^

**FIGURE 7:**
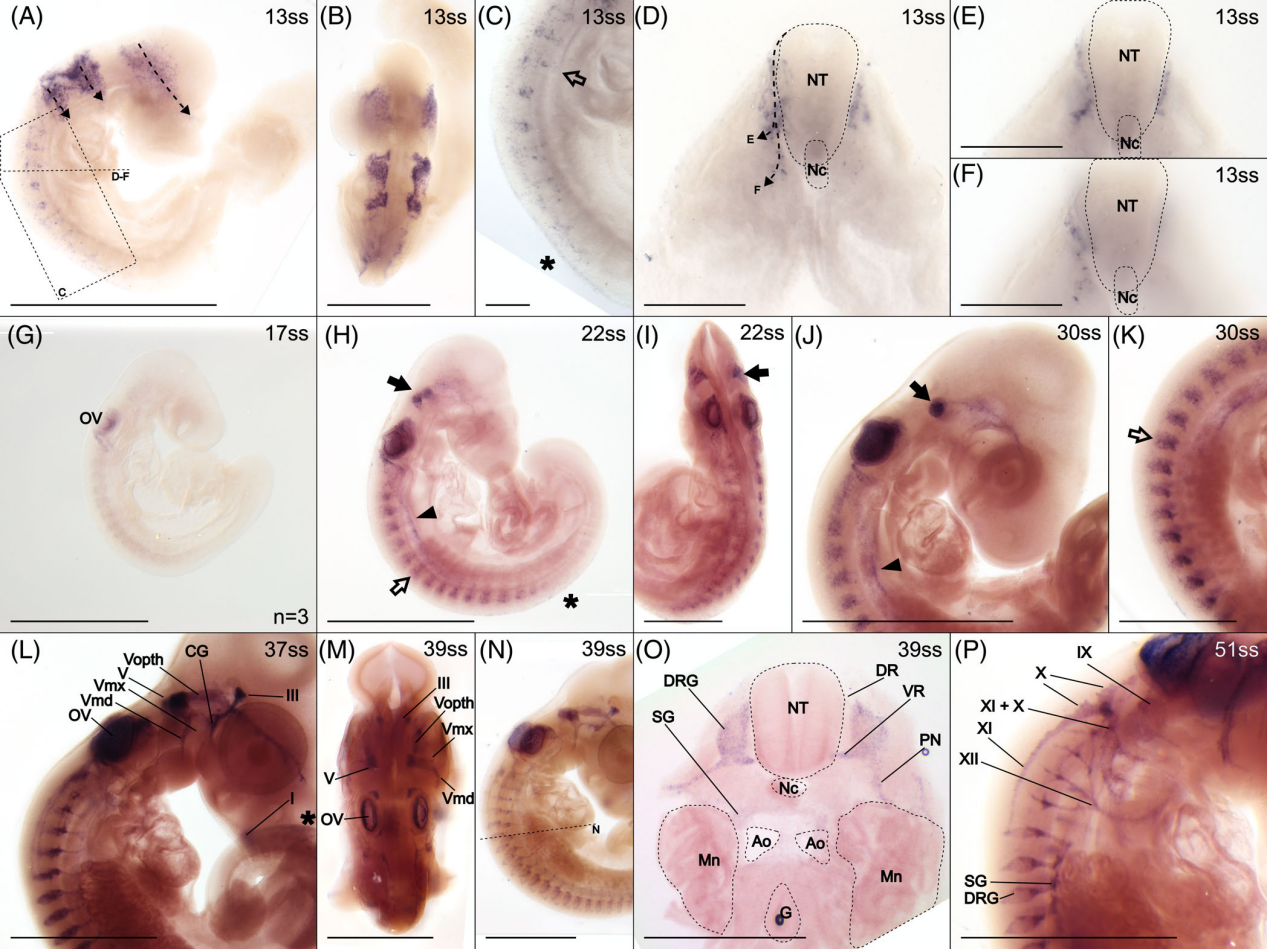
*Sox10* expression identifies migratory NCCs and the developing peripheral nervous system. Asterisks indicate premigratory and early migratory NCCs along the dorsal midline, arrows indicate the trigeminal nerve placode, open arrows indicate migratory trunk NCCs and arrowheads indicate caudally migrating vagal NCCs. (A)–(F) 13ss embryo in lateral (A,C) and dorsal (B) views and in cross‐section (D–F). The positions of (C)–(F) are indicated in (A). Dashed arrows indicate inferred streams of NCC migration. (D) is a composite of many focal planes, while (E) and (F) are single focal planes. (G) 17ss embryo in lateral view. Notice the weak signal, a result replicated in three separate experiments. (H,I) 22ss embryo in lateral (H) and dorsal (I) view. Head (J) and trunk (K) of 30ss embryo in lateral view. (L) 37ss embryo in lateral view. Asterisk indicates weak expression along the dorsal midline of the tail. (M–O) 39ss embryo in dorsal (M) and lateral (N) view, and in cross‐section (O). The approximate level of the cross‐section in (O) is indicated by a dashed line in (N). (P) 51ss embryo in lateral view. Ao, aorta; CG, ciliary ganglion; DR, dorsal root; DRG, dorsal root ganglion; G, gut; Mn, mesonephros; Nc, notochord; NT, neural tube; OV, otic vesicle; PN, peripheral nerve; SG, sympathetic ganglion; VR, ventral root. Cranial nerves: I, olfactory nerve (also known as cranial nerve 1); III, oculomotor nerve (also known as cranial nerve 3); V, trigeminal nerve (also known as cranial nerve 5); Vopth, ophthalmic branch of the trigeminal nerve; Vmd, mandibular branch of the trigeminal nerve; Vmx, maxillary branch of the trigeminal nerve; IX, glossopharyngeal nerve (also known as cranial nerve 9); X, vagus nerve (also known as cranial nerve 10); XI, spinal accessory nerve (also known as cranial nerve 11); XII, hypoglossal nerve (also known as cranial nerve 12). Scale bars in (B) and (O) are 0.5 mm and (C–F) are 0.1 mm. All other scale bars are 1 mm.

*Sox10* is expressed in all investigated stages (11 stages ranging from 13ss to 64ss; Figure 7A–P). All three streams of migratory cranial NCCs inferred from the HNK‐1 staining are clearly expressing *Sox10* (Figure 7A,B). However, *Sox10* expression, in contrast to HNK‐1, *Snai2*, *Snai1*, *Twist1*, and *Sox9* does not stain the streams all the way to the rostrum, maxillary process, and pharyngeal arches (Figure 7A). As development progresses, cranial *Sox10* expression, more distinctly than HNK‐1 staining, localizes in some cranial placodes (arrow in Figure 7H–J) and subsequently the corresponding nerves of the cranial peripheral nervous system (Figure 7L,M,P). A putative vagal stream migrating caudally in the trunk from the third cranial stream is marked by *Sox10* expression (arrowhead in Figure 7H,J). Later in development, the vagus nerve is clearly discernable through *Sox10* expression (Figure 7P).

Early *Sox10* expression in the trunk is largely similar to the spatiotemporal distribution of NCCs inferred from HNK‐1 staining including putative premigratory and migratory NCCs (Figure 7A,C,H,K). However, the expression in the putative premigratory NCCs along the dorsal midline is rather weak (asterisks in Figure 7C,H,K). In cross‐section at the level of the heart at 13ss, the inferred streams are revealed to migrate ventrally along the edge of the neural tube (Figure 7D–F). By looking at different levels along the anterior–posterior axis, two different routes of migration can be distinguished; one turns laterally and enters the somite (Figure 7D,E), presumably giving rise to dorsal root ganglia.^18^ The other one continues ventrally (Figure 7D,F), likely giving rise to sympathetic ganglia.^18^ The expression in the migratory streams subsequently condenses to a row of dorsal root ganglia in each somite with ventral projections to the much smaller ganglia of the sympathetic chain (Figure 7L). The dorsal root ganglia, including their dorsal and ventral roots and peripheral projections, are clearly visible in cross‐section, while sympathetic ganglia are evident more ventrally, though less strongly expressing *Sox10* (Figure 7O).

FoxD3 is an important transcription factor in NCC development.^17^ Early in NCC development, FoxD3 acts as an activator, allowing NCCs to delaminate from the neural tube, but later its role changes and it maintains the multipotency of NCCs by repressing differentiation.^18^, ^68^, ^69^

**FIGURE 8:**
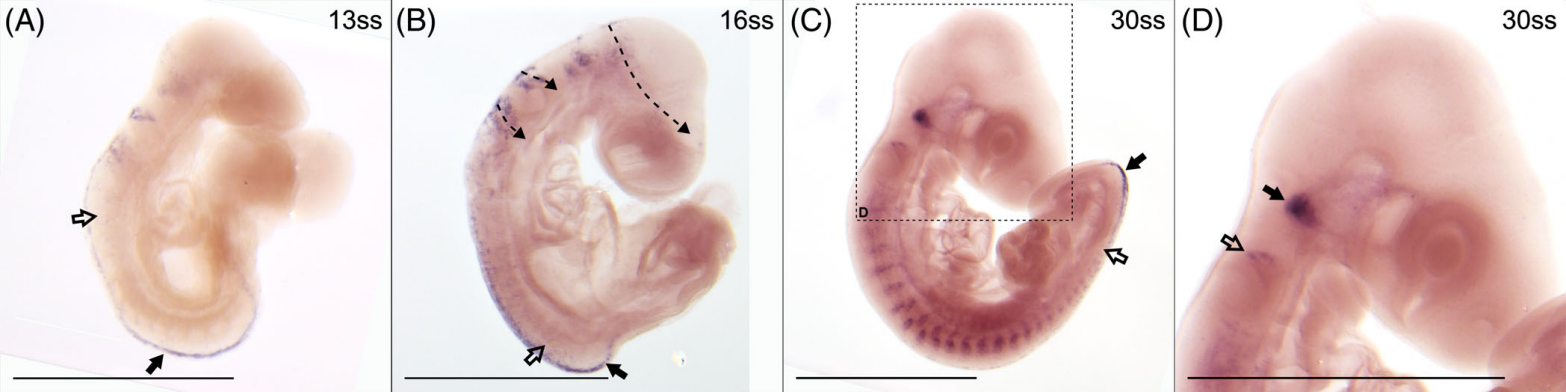
*FoxD3* expression labels of both premigratory and migratory NCCs as well as the developing peripheral nervous system. (A,B) 13 and 16ss embryos in lateral view. Dashed arrows indicate streams of migratory cranial NCCs, arrows indicate expression in premigratory NCCs, and open arrows indicate early migratory NCCs. (C,D) 30ss embryo in lateral view. Arrow indicates the trigeminal nerve placode and open arrow indicates the geniculate ganglion (also known as cranial nerve ganglion 7) The position of (D) is indicated in (A). Scale bars are 1 mm.

In the common wall lizard, *FoxD3* is expressed in all investigated stages (9 stages from 13ss to 64ss), and its expression patterns are similar to those of *Sox10*, although mostly not as strong. Like *Sox10*, *FoxD3* stain premigratory NCCs along the dorsal midline (Figure 8A–C), migratory NCCs including three cranial streams (Figure 8A,B), and NCCs condensing in the peripheral nervous system of both the head and trunk (Figure 8C,D). Unlike *Sox10*, *FoxD3* staining in premigratory NCCs along the dorsal midline is strong. It also stains in the geniculate ganglion (open arrow in Figure 8D) but not the otic vesicle.

**FIGURE 9:**
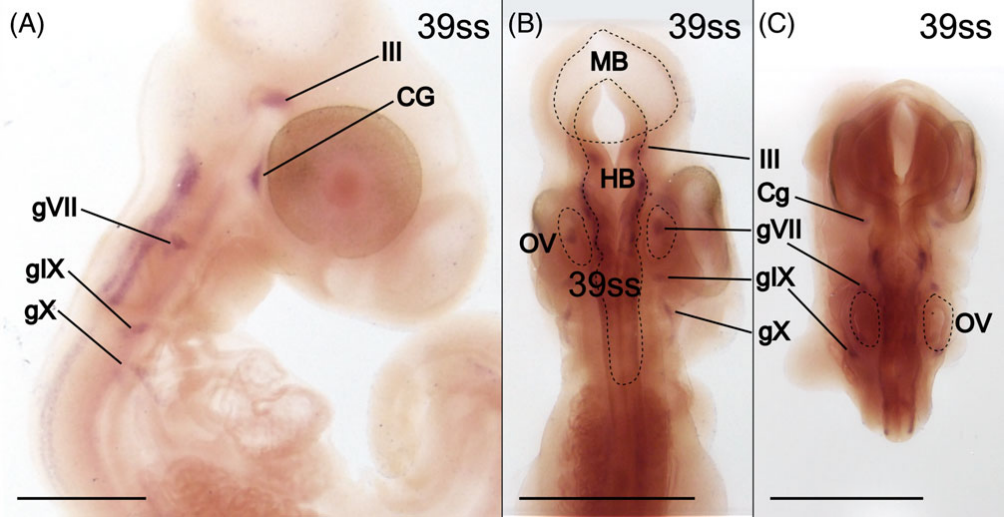
*Phox2b* expression identifies cranial ganglia. (A–C) Head of 39ss embryo in lateral (A) and dorsal (B,C) views. Expression signals in several cranial nerve ganglia are indicated. CG, ciliary ganglion; III, oculomotor nerve (also known as cranial nerve 3); gVII, geniculate ganglion (also known as cranial nerve ganglion 7); IX, glossopharyngeal ganglion (also known as cranial nerve 9); X, vagus nerve (also known as cranial nerve 10); OV, otic vesicle. Scale bars are 1 mm.

Downstream of *Sox10*, *Phox2b* is a regulator of NCC differentiation into autonomic and especially sympathetic neurons and chromaffin cell progenitors.^46^, ^70^, ^71^, ^72^ In the common wall lizard, *Phox2b* is expressed in all investigated stages (7 stages ranging from 17ss to 51ss) except the youngest (17ss; data not shown). However, the expression is most distinct at 39ss (Figure 9A–C), and expression before and after this stage is generally restricted and weak. *Phox2b* expression localizes to several of the cranial ganglia, which are co‐derived from the cranial placodes and NCCs.^73^ There is also significant expression in the hindbrain in the form of three longitudinal stripes on each side (Figure 9A), similar to previous results in mouse.^74^ Contrary to previous results in mouse and chicken, there is no distinct expression of *Phox2b* in the dorsal root ganglia in any of the investigated stages.^73^, ^74^

Taken together, gene expression profiles of NCCs biased toward neuronal and glial fates (*Sox10*, *FoxD3*, and *Phox2b*) largely confirm and expand the spatiotemporal distribution of NCCs inferred from HNK‐1 staining. Together their expression patterns clearly visualize the contribution of NCCs to the development of the peripheral nervous system, including the sympathetic nervous system.

### 2.5 *Th* expression identifies NCCs differentiating into chromaffin cells

In mouse, *Tyrosine hydroxylase* (*Th*) has been identified as a suitable marker for separating the chromaffin cells from the sympathetic neurons.^71^, ^75^ It is an enzyme catalyzing one of the reactions in the metabolic pathway that result in the production of adrenalins.^76^

**FIGURE 10:**
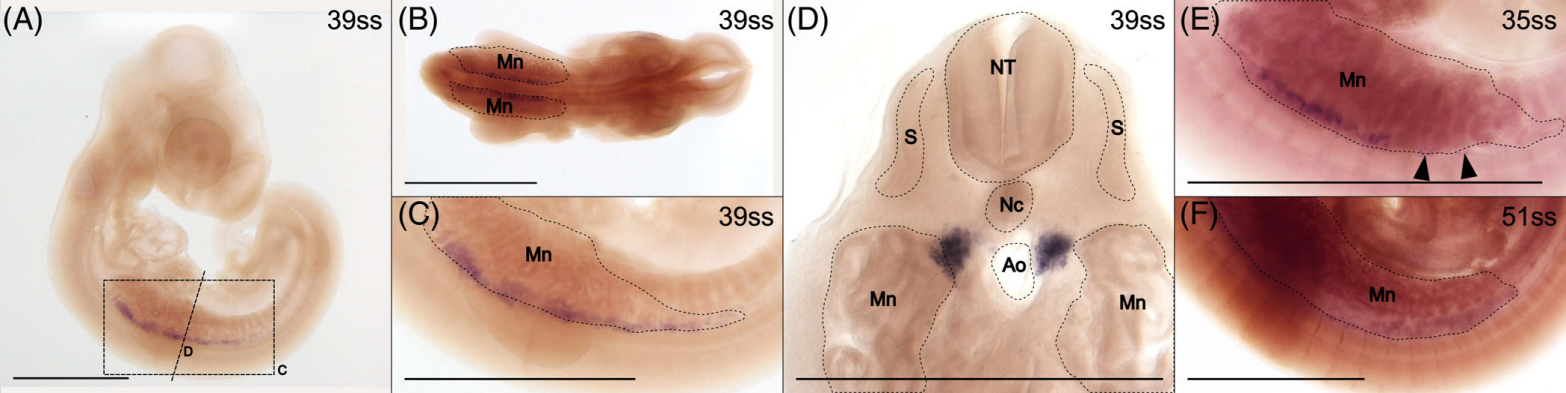
*Th* expression identifies the chromaffin cells of the putative adrenal gland. (A–D) 39ss embryo in lateral (A,C) and dorsal (B) view and in cross‐section (D). Dashed rectangle and line in (A) indicate the approximate position of panels (C) and (D), respectively. (E,F) Trunk of 35 and 51ss embryos in lateral view. Arrowheads indicate discontinuity in the expression. Ao, aorta; Mn, mesonephros; Nc, notochord; NT, neural tube; S, somite. Scale bar in (D) is 0.5 mm, all others are 1 mm.

*Th* expression is first detected at 35ss and is maintained at least until 51ss. The expression is located internally in the trunk, at the level of the forelimb bud (Figure 10A–F). *Th* expression is localized in two lateral parallel lines (Figure 10B) in the dorsomedial corner of the mesonephros (Figure 10D). This is putatively the position where the sympathetic ganglia including the adrenal glands are developing. The areas of expression are discontinuous (see for example arrowheads in Figure 10E) concordant with chromaffin cells being restricted to the sympathetic ganglia including the adrenal glands.

## 3 CONCLUSIONS

**FIGURE 11:**
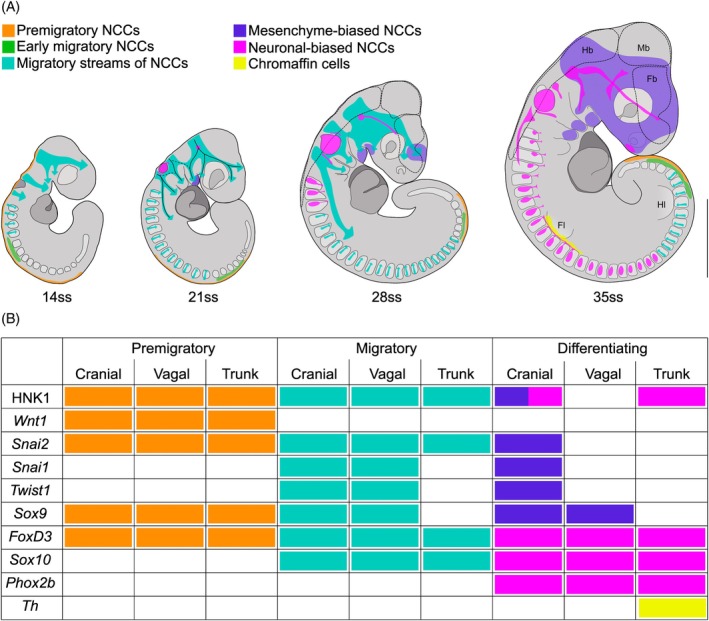
Synthesis of the spatiotemporal distribution of NCCs in developing common wall lizards. (A) Summary of the inferred distribution and migratory streams of NCCs in four developmental stages of the common wall lizards. Different groups of NCCs are color‐coded according to their state or expected fate. (B) Schematic overview of the state of NCCs (premigratory, migratory, or differentiating) and their respective domain (cranial, vagal, or trunk) with respect to their expression of individual marker genes and the presence of HNK‐1 positive staining. Fb, forebrain; Fl, forelimb; Hb, hindbrain; Hl, hindlimb; Mb, midbrain.

Taken together, the insights gained from HNK‐1 immunostainings and gene expression patterns of nine marker genes provide a detailed profile of the spatiotemporal distribution of NCCs in developing wall lizard embryos (Figure 11). By the time of egg‐laying, the embryo has already reached at least 13ss at which extensive migration is ongoing in the head. Around 35ss, most migration is completed and differentiation toward various fates is well underway. In the following, we briefly summarize the major patterns of NCC development at four different stages.

At 14ss, premigratory NCCs are present along the dorsal neural tube all the way from the midbrain–hindbrain boundary to the last formed somite pair (orange in Figure 11A). Streams of cranial NCCs are already migratory. In the caudal trunk, there is no migration observed at this stage. At more anterior regions in the trunk, a field of NCCs lateral to the dorsal midline indicates that the first wave of NCC migration has started but these cells have not yet entered the somites (green in Figure 11A). In the anterior‐most part of the trunk, migration has proceeded further with the first migratory streams traversing the anterior parts of the somites (turquoise in Figure 11A).

At 21ss, NCC specification and migration in the trunk has proceeded further posteriorly and migration now occurs through several of the anterior‐most somites (approximately the first 13–15). In the head, the streams of migratory cranial NCCs have reached further and are bifurcating more. NCCs with mesenchymal fate are located in the first pharyngeal arch (purple in Figure 11A), and NCCs with neural fate are located in the otic vesicle and trigeminal nerve placode (magenta in Figure 11A). Vagal NCCs appear as a branch extending caudally through the anterior trunk from the third cranial stream.

By 28ss, NCC specification and migration in the trunk has proceeded even further posteriorly and the first differentiated dorsal root ganglia occur in the anterior‐most somites (approximately the first 3; magenta in Figure 11A). The cranial streams are now more advanced. NCCs with mesenchymal fate appear in the maxillary process and the rostrum and NCCs with neural fate are expanding in cranial placodes and the first neural projections (magenta in Figure 11A).

At 35ss, specification and migration of NCCs is restricted to the caudal‐most trunk region. Neuronal differentiation is well underway in both the dorsal root ganglia and the sympathetic ganglia of the trunk. At the level of the forelimb, some NCCs have differentiated into chromaffin cells (yellow in Figure 11A). The mesenchymal NCCs are now distributed broadly in the head including the first three pharyngeal arches, maxillary process, rostrum, and close to the olfactory pit. Cranial nerves have developed further: the olfactory (I), oculomotor (III), trigeminal (V), glossopharyngeal (IX), vagus (X), and spinal accessory nerves are now all clearly distinguishable.

This synthesis is derived from the collective information gathered from ten markers (Figure 11B), which proved to be crucial to overcome incomplete information stemming from individual markers. For example, HNK‐1 immunostaining and *Phox2b* expression signals are not located in the dorsal root ganglia, yet other markers, for example, *Sox10* and *FoxD3*, clearly identify this tissue as NCC‐derived. The HNK‐1 signal in dorsal root ganglia reported for the veiled chameleon^9^ is also less distinct than typically seen in other species. Thus, the lack or reduction of HNK‐1 on dorsal root ganglia may be a more widespread feature of lizards. In addition, since there are no distinct NCC markers that identify exclusively NCCs, off‐target staining necessarily needs to be taken into account. Examples include the expression of *Snai1* and *Twist1* in trunk mesoderm or the presence of HNK‐1 on blood cells.

Comparing our synthesis of NCCs in the common wall lizard to those reported for other squamates (the Californian kingsnake,^7^ Egyptian cobra,^8^ and veiled chameleon^9^) reveals that NCC biology is largely conserved. One caveat for this comparison is that our analysis is restricted to embryos older than 13ss, and we therefore lack observations of the earliest stages of NCC specification and migration, particularly in the head. It was therefore not possible to investigate if common wall lizards, similar to chameleons^9^ and crocodiles,^16^ possess an early rostral stream of migrating cranial NCCs stemming from the level of the forebrain. In future studies, efforts could be made to obtain earlier embryos by, for example, treating gravid females hormonally to induce premature egg laying,^77^ or by selecting lizard species that lay eggs at earlier developmental stages.

The two main exceptions of NCC regulation in squamates relative to other tetrapods that have been reported to date are the fourth stream of migrating NCCs from the hindbrain observed in the veiled chameleon^9^ (also found in an alligator and an ostrich)^16^ and the late wave of trunk NCCs taking a ventromedial (instead of the expected dorsolateral route), which was found in the Californian kingsnake.^7^ Our observations in the common wall lizard do not clearly identify a fourth stream of NCCs from the hindbrain. However, to confidently rule out the existence of this stream, further analyses including cell lineage tracing may be required. Regarding the ventromedial route of late wave trunk NCCs, we do find ventromedial intra‐somitic migration that is coinciding with dorsolateral migration, but it is not clear that these correspond to the late wave ventromedial migration reported for the California kingsnake.^7^ On a more technical note, it would also be desirable to confirm and cross‐validate results in various species with the same antibody given that observed species‐level differences might be partially attributed to the use of different antibodies targeting HNK‐1.

In summary, our study provides the first complete description of NCCs, including cranial, vagal, and trunk NCCs, in a lacertid and contributes to our understanding of taxon‐specific differences in the dynamic distribution of this cell type. Thus, it lays the foundation for future work targeting the role of this cell type in evolutionary diversifications.

## 4 EXPERIMENTAL PROCEDURES

### 4.1 Embryo collection

All embryos stem from captive breeding colonies of common wall lizards that were wild caught in central Italy. Parents showed variation in phenotypes (coloration, morphology, and behavior), including in traits derived from the neural crest. In the present study, we describe broad developmental patterns that we do not expect to differ on a population level, and we therefore do not discriminate between embryos of different parental origins.

Adult wall lizards were kept as described in Feiner et al., (2018).^78^ Eggs were collected during the breeding seasons (April–July) of 2021–2023. Each cage had a pot filled with moist sand in which females laid their clutches. The eggs were collected each morning, and one egg per clutch was dissected immediately to determine the developmental stage (somite count) of the embryos in the clutch. The rest of the eggs were incubated at 24°C in moist vermiculite until the appropriate stage was reached. We aimed to sample a distribution of stages from as young as possible up to around 60ss. Based on 563 eggs, the mean embryonic stage on the day of oviposition was estimated to be 21.4ss (sd = 5.2 somites) and the earliest embryos were at 11ss. The pace of somite formation is constant and has previously been estimated to be four somites per day when incubated at 24°C.^78^

Egg dissections were performed in cold, nuclease‐free PBS (10 mmol/L phosphate buffer, 2.7 mmol/L KCl, and 137 mmol/L NaCl, pH 7.4) after which each embryo was immediately transferred to 4% paraformaldehyde in PBS for overnight fixation at 4°C on a rotating platform. After fixation, embryos were transferred to gradually increasing concentrations of methanol and stored in 100% methanol at −20°C. A small subset of embryos intended to be used in probe synthesis (see below) was dissected as described above, incubated in RNAlater (Invitrogen AM7020) at 4°C for 24 h and then transferred to −80°C for storage.

### 4.2 Immunohistochemistry

Paraformaldehyde‐fixed embryos were rehydrated in PBS‐T with decreasing concentrations of methanol. Embryos were blocked using TN‐blocking buffer with bovine serum albumin (1% bovine serum albumin, 0.1 mol/L Tris pH 7.5, 0.15 mol/L NaCl in H_2_O) followed by overnight incubation with primary antibodies against HNK‐1 (1:500; Anti‐Hu CD57 eBioscience 11‐057742) in TN‐blocking buffer. Embryos were washed with PBS and incubated overnight with secondary antibodies (1:200; Alexa Flour 488 goat anti‐mouse IgM [μ chain] Invitrogen A21042) in TN‐blocking buffer. Embryos were washed in PBS and stained with DAPI (1:500; ThermoScientific 62,248). Lastly, embryos were cleared by overnight incubation in RapiClear 1.49 (SUNJin Lab) and then mounted in fresh RapiClear medium between two 0.17 μm cover slips using iSpacers (SUNJin Lab). The stained and cleared embryos were imaged using a confocal microscope (Leica TCS SP8) using a 20×/0.75 IMM objective (plan apochromatic multi‐immersion). Tiles were merged and 2D images were rendered using the LAS X software version 0.5.7.23225.

### 4.3 Probe synthesis for in situ hybridization

Total RNA was extracted from the RNAlater‐stored embryos using the RNeasy Mini Kit (Qiagen) including DNase digestion and stored in nuclease‐free water at −80°C. Extracted RNA was used as a template in reverse transcription into cDNA using SuperScript III Reverse Transcriptase and oligo‐dT primers following the manufacturer's instructions. The selected marker genes were amplified by polymerase chain reaction (PCR) from the cDNA using GoTaq G2 Hot Start Green Master Mix (Promega), and primers designed using the PodMur_1.0 reference genome.^79^ Primers were designed to amplify 800–1200 bp of the gene of interest with ~500 bps in the 3′UTR and T7 and SP6 promoters attached to the reverse and forward primers, respectively (see Table 1 for primer sequences). Amplified gene fragments were Sanger‐sequenced to confirm each amplicon's identity. PCR products were purified using the MinElute PCR Purification Kit (Qiagen) and in vitro transcribed to produce antisense RNA probes using the DIG Probe Synthesis Kit (Roche). The resulting RNA probes were purified using the RNeasy Mini Kit (Qiagen) and stored in nuclease‐free water at −80°C.

**TABLE 1:** Sequences of primers used in this study (5′→3′).

Gene	Forward primer	Reverse primer
*Snai2*	ATAAGCAGTTGCACTGTG	GTGTGTATCAAGCATGTG
*Eya2*	GATGGTGTTGAAGAGGAA	AGGTAACTCTTTCTCCTG
*FoxD3*	AAAGCCTTCAACTCCCAG	CTCCCAATCCTTTGCTTG
*Snai1*	AACTGTGAGAAGGAGTAC	CAAGAGAGAATTGAGTGG
*Sox9*	AGTTTGACCAGTATCTCC	TAAGGCATGCCATTTTGC
*Sox10*	AAAGACAGAACTGCAGTC	GAGGGTCGTATATACAG
*Th*	ATAATATCCCTCAGCTGG	TGGATAGCAACACAAACC
*Twist1*	TACATCGACTTCCTCTAC	AAATGAAAGAGGAGCAGG
*Wnt1*	CTCTGCAAGAATAGGAAC	TCATCTACGGCAACAAAG

### 4.4 Whole mount in situ hybridization

Following the methodology in Feiner (2019),^80^ paraformaldehyde‐fixed embryos were rehydrated in nuclease‐free PBS‐T (1× PBS with 0.1% Tween‐20) using gradually lower concentrations of methanol. Embryos were permeabilized using Proteinase K, followed by refixation using 4% paraformaldehyde in PBS. Hybridization was done with 100 ng/mL probe at 68°C in hybridization buffer (5× SSC pH 7.5, 50% deionized formamide, 50 μg/mL heparin, 0.3 mg/mL torula RNA, 2% Tween‐20, 2% blocking reagent, and 10 mmol/L EDTA). Hybridized embryos were blocked in blocking buffer (1.5% blocking reagent in PBS‐T), followed by incubation with 0.1875 U/mL anti‐DIG antibody in blocking buffer. This was followed by 1.5 days of washes in maleic acid buffer (15 mmol/L maleic acid pH 7.5, 300 mmol/L NaCl, and 0.1% Tween‐20) before color development with 2% NBT/BCIP in alkaline phosphatase buffer (0.1 mol/L Tris Base pH 9.5, 0.1 mol/L NaCl, 1 mmol/L MgCl_2_, and 0.1% Tween‐20). Once a good signal‐to‐noise ratio was achieved, the reaction was stopped by extensive washes in PBS‐T and re‐fixing in 4% paraformaldehyde. Some stained embryos were cross‐sectioned by hand using a scalpel. Stained embryos and sections were photographed in glycerol using a Nikon SMZ18 stereo microscope with a Nikon SHR Plan Apo 2X WD: 20 objective and Nikon DS‐Ri1 camera.

**Table:** 

Reagents List	Company	Catalog#	cas# (Chemical Abstracts Service Number)
PBS	Sigma	P4417	
Water, nucl. free, Mol. Biol. Grade, Ultrapure	ThermoScientific	J71786.K8	
Paraformaldehyde	Sigma‐Aldrich	P6148	30525‐89‐4
Methanol	Merck Millipore	1.06009.1000	67‐56‐1
RNAlater	Invitrogen	AM7020	
Tween‐20	Sigma	P9416	
Tris Base for molecular biology white crystals or powder	Fisher Scientific	BP152	77‐86‐1
Sodium chloride, molecular biology grade (NaCl)	MP	194848	7647‐14‐5
Bovine serum albumin (BSA)	Sigma	A9418	
Primary antibody: CD57 monoclonal antibody (TB01 [TBO1]), FITC	eBioscience	11‐0577‐42	
Secondary antibody: Alexa Fluor 488 goat anti‐mouse IgM (u chain)	Invitrogen	A21042	
DAPI	ThermoScientific	62248	
RapiClear 1.49	SUNJin Lab	RC149001	
Cover glass 1.5 (~0.17 μm)	VWR	631‐0134	
iSpacer	SUNJin Lab		
RNeasy Mini Kit	Qiagen	74104	
SupersScript III reverse transcriptase	Invitrogen	18080	
GoTaq G2 hot start green master mix	Promega	M742A	
MinElute PCR purification kit	Qiagen	28004	
PCR DIG Probe Synthesis Kit	Roche	11636090910	
Proteinase K, recombinant PCR Grade	Roche	3115879001	
20× SSC	Fisher BioReagents	BP13254	
Formamide deionized for mol. biology	PanReac AppliChem	A2156	1975‐12‐07
Heparin sodium salt from porcine intestinal mucosa	Sigma‐Aldrich	H4784	9041‐08‐01
Riobonucleic acid from torula yeast	Sigma	R6625	63231‐63‐0
Blocking reagent	Roche	11096176001	
EDTA	Invitrogen	AM9260G	
Anti‐digoxenin‐AP Fab fragments	Roche	11093274910	
Maleic acid	Sigma‐Aldrich	M0375	110‐16‐7
NBT/BCIP	Roche	11681451001	
Glycerol	VWR	24397.3	56‐81‐5

### 4.5 Gene orthology

**FIGURE 12:**
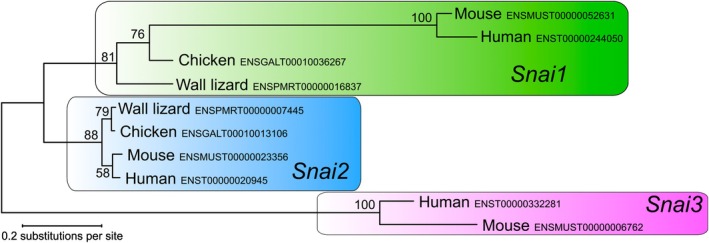
Gene tree of *Snai1*, *‐2* and *‐3*. Orthology relations of the transcription factors *Snai1* and *‐2* in the common wall lizard was confirmed with regard to human, mouse, and chicken. The *Snai3* gene appears to be absent from the common wall lizard genome. Accession numbers from Ensemble (wall lizard) and NCBI (mouse, human, and chicken) are given next to each branch tip.

Since the orthology relationships of *Snai1* and *Snai2* to other tetrapod genes was unclear,^61^ a gene tree was constructed to confirm their identities. Both sequences from the common wall lizard were obtained from Ensemble and blasted (protein blast) against the human, mouse, and chicken databases on NCBI, and the top hits were retrieved. Alignment (using the muscle algorithm), model selection (JTT+G), and maximum likelihood tree construction were performed in MEGA (Figure 12).^81^

## FUNDING INFORMATION

This work was supported by the Kungliga Fysiografiska Sällskapet i Lund (42010), the Jörgen Lindströms stipendiefond to RP, the European Research Council (948126), and the Swedish Research Council (2020‐03650) to NF.

## CONFLICT OF INTEREST STATEMENT

The authors of this study declare no conflicts of interest.
